# SERS Detection of Environmental Variability in Balneary Salt Lakes During Tourist Season: A Pilot Study

**DOI:** 10.3390/bios15100655

**Published:** 2025-10-01

**Authors:** Csilla Molnár, Karlo Maškarić, Lucian Barbu-Tudoran, Tudor Tămaș, Ilirjana Bajama, Simona Cîntă Pînzaru

**Affiliations:** 1 National Institute for Research and Development of Isotopic and Molecular Technologies, 67-103 Donath, 400293 Cluj-Napoca, Romania; 2 Biomolecular Physics Department, Babeş-Bolyai University, Kogălniceanu 1, 400084 Cluj-Napoca, Romania; karlo.maskaric@ubbcluj.ro (K.M.); ilirjana.bajama@ubbcluj.ro (I.B.); 3 Institute for Research, Development and Innovation in Applied Natural Sciences, Babeş-Bolyai University, Fantanele 30, 400327 Cluj-Napoca, Romania; 4 Electron Microscopy Centre, Babeș-Bolyai University, Clinicilor 5-7, 400006 Cluj-Napoca, Romania; lucian.barbu@itim-cj.ro; 5 Geology Department, Babeş-Bolyai University, Kogălniceanu 1, 400084 Cluj-Napoca, Romania; tudor.tamas@ubbcluj.ro

**Keywords:** Raman, SERS, salt lakes monitoring, in situ, seasonal fluctuations

## Abstract

This pilot study uses Raman spectroscopy and SERS to monitor monthly water composition changes in two adjacent hypersaline lakes (L1 and L2) at a balneary resort, during the peak tourist season (May–October 2023). In situ pH and electrical conductivity (EC) measurements, along with evaporite analyses, complemented the spectroscopic data. Although traditionally considered similar, the lakes frequently raise public questions about their relative bathing benefits. While not directly addressing the therapeutic effects, the study reveals distinct physicochemical profiles between the lakes. Raman data showed consistently higher sulfate levels in L2, a trend also observed in winter monitoring. pH levels were higher in L1 (8–9.8) than in L2 (7.2–8), except for one October depth reading. This trend held during winter, except in April. Surface waters showed more variability and slightly higher values than those at 1 m depth. SERS spectra featured β-carotene peaks, linked to cyanobacteria, and Ag–Cl bands, indicating nanoparticle aggregation from inorganic ions. SERS intensity strongly correlated with pH and EC, especially in L2 (r = 0.96), suggesting stable surface–depth chemistry. L1 exhibited more monthly variability, likely due to differing biological activity. Although salinity and EC were not linearly correlated at high salt levels, both reflected seasonal trends. The integration of Raman, SERS, and physicochemical data proves effective for monitoring hypersaline lake dynamics, offering a valuable tool for environmental surveillance and therapeutic water quality assessment, in support of evidence-based water management and therapeutic use of salt lakes, aligning with goals for sustainable medical tourism and environmental stewardship.

## 1. Introduction

Exploring the therapeutic and environmental benefits of hypersaline lakes has attracted considerable scientific interest, as these unique ecosystems are characterized by high salinity, diverse microbial communities, and intricate biochemical processes that confer distinctive physicochemical properties [1]. Such features may underlie their medical recommendation for bathing cure in various skin, muscular, metabolic, or rheumatic diseases, as well as for wellbeing and stress relief [2,3,4]. In Cluj County, Transylvania, Romania, the Cojocna Balneary Resort hosts two notable hypersaline lakes—Török Lake (L1) and the Great Lake (L2)—which originated through the collapse of former salt mines later filled with water [5,6]. Their location, depth profiles, and initial physicochemical and spectroscopic characteristics have been recently reported in a six-month monitoring program [7] that focused on the upper to 1 m water layer, suitable for bathing, investigated monthly during the cold season when touristic activity is minimal. These anthropic salt lakes reflect the region’s long history of salt exploitation, shaped by “bell” or “trapezoidal” mining systems in the diapiric area along the Transylvanian Plateau’s border. Over time, precipitation and underground sources accumulated in the abandoned mines, fostering subsurface interconnections. The Cojocna lakes complex remains closely linked to the historical saltworks, which were abandoned in the mid-19th century (1850–1852) [2,3,4,5,6,7]. Therapeutic use of cold, salty waters began in 1883 with the founding of the Cojocna station, initially centered on Băilor Lake, which was soon developed as a spa attraction [2].

Among the various analytical techniques used to study such environments [8], surface-enhanced Raman Spectroscopy (SERS) [7,9] has emerged as a powerful tool for detecting and characterizing molecular compositions with high sensitivity and specificity. In our previous monitoring pilot study of the hypersaline lakes L1 and L2 of Cojocna carried out during winter months (October to April) using SERS of salt waters [7], combined with Raman spectroscopy of raw waters and measured parameters (temperature, pH, electrical conductivity), we found significant spectral and chemical differences between waters from the two adjacent hypersaline lakes. Raman and SERS data clearly distinguished their organic and inorganic compositions, as well as their temporal variations. These observed changes, which correlate with the spectroscopic and physicochemical properties of hypersaline waters, are particularly relevant not only for the correct management of the resort, but also for ongoing monitoring needs, either for balneotherapy purposes or for tracking the dynamics of the salt bedrock and subtle changes in surface water level and composition.

Here, we conducted a six-month summer monitoring initiative (May–October), measuring both the physicochemical and spectroscopic properties of hypersaline lake waters during the high touristic season. This manuscript provides additional insights into the applicability of SERS for monitoring such environments by combining spectroscopic data with physicochemical parameters, providing scientific knowledge that is requested in the context of currently renowned balneary resort facilities, aiming to provide high-quality services and scientifically based evidence of water properties. Regular monthly measurements were conducted over the summer months, when the anthropogenic influence is high due to the bathing season and when the climate conditions are radically different compared to those from the winter monitoring period [7]. This study provides the first analytical insight into the temporal variations in the lake waters’ molecular composition, monitored monthly from May to October, when touristic activities are dominant in the area. Correlations between physicochemical parameters and spectroscopic data were assessed, comprising both Raman spectroscopy of surface waters and their corresponding SERS data, which illustrated the inorganic–organic counterpart.

Sulfate level readily measurable via normal Raman spectroscopy of waters [10] is expected to provide linkage with the overall salinity, which in turn determines the specific aggregation of silver nanoparticles (AgNPs) used for SERS measurements of waters [7]. These links and correlations are explored from the physicochemical and spectroscopic data, aiming to provide insight into the inorganic–organic content of the salt waters during balneary exploitation. These findings raise important questions regarding the degree to which seasonal and anthropogenic variables impact the water chemistry and molecular composition of hypersaline lakes. Therefore, we hypothesize that the intensified human activity during the summer bathing season may significantly alter the spectroscopic signatures and physicochemical balance of these ecosystems. To test this hypothesis, we conducted monthly field sampling from May to October and applied both classical physicochemical analyses and Raman/SERS spectroscopic techniques to monitor the dynamics of dissolved inorganic and organic constituents. Beyond the scientific insights, our results aim to support the implementation of evidence-based water management practices and therapeutic guidelines for natural salt lakes under active balneary exploitation. This is particularly relevant in the context of increasing interest in sustainable medical tourism and environmental stewardship of mineral-rich water resources.

## 2. Material and Methods

### 2.1. Field Sampling of Waters

Monthly, triplicate, raw water samples were collected from each of the two neighboring salt lakes designated L1 and L2 following a similar protocol as for the previous pilot study of waters in winter months [7]. Each lake contributed one sample from a depth of 1 m and two samples from surface waters (approximately 15 cm depth) during the warm season spanning from May 2023 to October 2023, resulting in a total of 36 raw water samples (2 lakes × 3 samples per lake × 6 months). Given the high salinity, only the upper water layer (first meter) was considered, as it is the sole accessible stratum for balneary bathing purposes.

Water pH, temperature, and electrical conductivity (EC) were immediately measured in situ. Lake water was then carefully collected into 500 mL vials (three per lake), promptly transported to the laboratory, and stored under cold and dark conditions. Simultaneously, subsamples were prepared for Raman and SERS immediate measurements, all conducted on the same day as collection. Permission for experimental sampling was granted via a collaboration agreement with the local authorities managing the Cojocna Balneary Lakes.

### 2.2. Sample Preparation for Raman and SERS Measurements

For micro-Raman spectra, liquid water samples were analyzed using the Drop Coating Deposition Raman (DCDR) technique. Specifically, 10 µL droplets from each water sample were deposited onto a hydrophobic, Teflon-coated stainless steel µ-RIM-TM slide from BioTools (Jupiter, FL, USA). SERS samples were prepared by adding 10 µL of raw water samples to 500 µL of silver colloidal solution and then measured in 2 mL glass vials with caps. AgNP stocks were freshly prepared at the beginning of the study and carefully preserved in dark and frigid conditions for SERS monthly monitoring experiments on lake waters. The acquisition time was 10 s with one scan, and the laser power was 100 mW.

Salt samples were obtained from each sampling water batch and allowed to evaporate in room conditions on the same day as water collection. Precisely 20 mL of aliquots of lake water from both L1 and L2, collected at a depth of 1 m, was weighed and transferred into clean glass containers. To prevent contamination, each sample was covered with filter paper and allowed to evaporate at room temperature (20 °C). After complete drying, the remaining salt residues were weighed to determine the total mass of dissolved salts (organic counterpart included) originally present in the water. These values were then extrapolated to express the total salt content in grams per liter (g/L) of lake water.

### 2.3. Silver Colloid Synthesis and Characterization

Silver colloids were synthesized following the classical Lee and Meisel protocol [11]. Materials included silver nitrate and sodium citrate; both were purchased from Sigma-Aldrich, St. Louis, MO, USA. The resulting colloid (silver nanoparticles (AgNPs)) was characterized immediately using UV-VIS absorption spectroscopy, showing a surface plasmon resonance (SPR) peak centered at 417 nm, indicative of AgNP formation. The resulting AgNP stock was freshly characterized using transmission electron microscopy (TEM), and its typical morphological features and size distribution (Supplementary Information, Figure S1) were found to be fully consistent with previously reported and commonly used AGNP stocks [7]. The SERS activity of the nanoparticles was further confirmed by preliminary tests on well-known molecules for SERS, such as methylene blue and crystal violet.

### 2.4. X-Ray Diffraction (XRD) Analysis of Salt Residues

To evaluate the mineralogical composition of the precipitated salts, 1 L surface water samples from each lake (L1 and L2) were left to evaporate at room temperature in clean, dust-free conditions. After complete evaporation, the resulting crystalline salt residues were collected and ground to a fine powder for analysis.

### 2.5. Instrumentation

In situ measurements of EC and pH were performed using HQ40d multiparameter equipment (HACH, Loveland, CO, USA). The conductivity resolution ranged from 0.01 µS/cm to 0.1 mS/cm, and the pH resolution was adjustable to 0.001, 0.01, and 0.1 pH units.

Micro-Raman spectra were acquired using a Renishaw InVia Reflex confocal Raman system (Gloucestershire, UK). Excitation was provided by a 532 nm Cobolt diode-pumped solid-state (DPSS) laser (100 mW), focused through a Leica confocal microscope with a 5× objective. Spectra were collected with a RenCam CCD detector (Renishaw, Gloucestershire, UK, 1024 × 256 pixels, 0.5 cm^−1^ spectral resolution).

UV-VIS absorbance spectra were recorded using a Shimadzu 1900 spectrophotometer (Shimadzu, Kyoto, Japan), scanning from 200 to 1100 nm in a 1 nm step size.

SERS spectra were obtained using a portable Wasatch Photonics WP 532-A -SR- IC modular Raman spectrometer (Wasatch Photonics, Morrisville, NC, USA), with a 532 nm laser (10 mW output). Measurements were performed in triplicate per sample over the 100–3200 cm^−1^ spectral range, with a resolution of 14 cm^−1^. All measurements were performed at room temperature.

Scanning electron microscopy (SEM) was used to investigate the morphology of evaporated salt residues and microorganisms inhabiting the water. Analyses were performed using a Hitachi SU8230 cold field emission scanning transmission electron microscope (Hitachi, High-Tech Corporation, Tokyo, Japan).

X-ray diffraction (XRD) measurements of bulk evaporated salt residues were performed for mineralogical phase identification using a Bruker D8 Advance diffractometer operating in Bragg–Brentano geometry with a Cukα radiation source (λ = 0.15418 nm), a Ni filter, and a LynxEye position-sensitive detector, enabling high-resolution pattern acquisition and reliable phase identification. Corundum (NIST SRM1976a) was employed as an internal standard for calibration. Scans were collected over the 3.8–64° 2θ range, using a step size of 0.02° 2θ and a counting time of 0.2 s per step. Phase identification was conducted using the Diffrac Eva 2.1 software (Bruker AXS GmbH, Karlsruhe, Germany) in combination with the PDF2 (2012) database.

### 2.6. Data Processing and Statistical Analysis

Data processing was performed using OriginPro 2021b (OriginLab Corporation, Northampton, MA, USA). Averaged raw Raman spectra were calculated from three independent subsamples per case and used for subsequent analytical evaluation. For surface waters, the spectra obtained from the two diametrically opposite sampling locations on each lake were averaged and combined with an additional spectrum recorded from the 1 m depth sample, resulting in two spectra per lake per month. This design yielded a total of 24 datasets (2 lakes × 2 composite samples × 6 months) for statistical analysis.

To explore the correlation between spectral data and physicochemical properties, Pearson correlation coefficients [12] were calculated using Python version 3.6.3 in the Anaconda-JupyterLab environment. The following Python libraries were employed: pandas, NumPy, matplotlib.pyplot, seaborn, and scipy.stats (specifically, the pearsonr function).

For Raman spectra of waters, the band intensity at 981 cm^−1^, corresponding to the sulfate stretching mode [9,13], was used as a representative inorganic marker. For SERS spectra, the strong signal at 245 cm^−1^, attributed to Ag–Cl interactions due to chloride-induced aggregation of AgNPs, was selected as a key indicator of the inorganic profile. Additionally, the SERS band at 1512 cm^−1^, assigned to the C=C stretching of β-carotene [14,15], was used as a marker for organic, microorganism-derived signatures.

pH and EC values, measured in triplicate at the sampling sites, were averaged for each lake and date of collection, and correlated with the corresponding Raman/SERS intensity values. All statistical evaluations were based on raw Raman spectral datasets, without baseline correction or normalization. However, SERS spectra were additionally processed for comparative visualization purposes by applying background subtraction and min-max normalization to a [0, 1] scale.

## 3. Results

### 3.1. Physical and Chemical Characteristics of Surface Waters from Hypersaline Lakes

**Figure 1:**
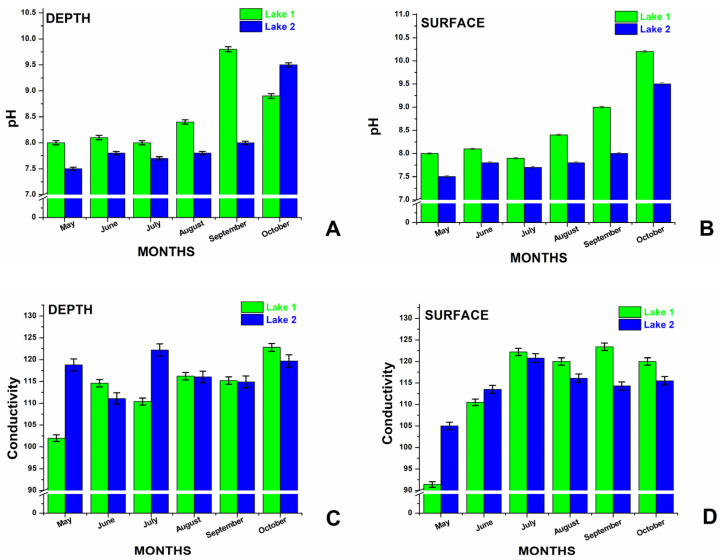
Comparative plot of the pH and electrical conductivity determined in situ for the water of the hypersaline lakes denoted L1 and L2, as indicated, at 1 m depth (panels (**A**,**C**)) and at the surface (panels (**B**,**D**)) during the summer season (May–October 2023). Error bars indicate ±0.5% for pH and ±0.85% for conductivity.

**Figure 2:**
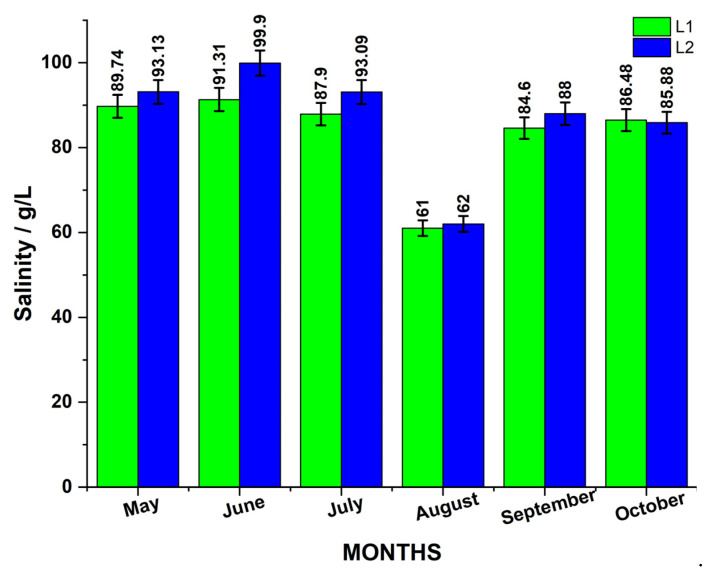
Monthly determined salinity level of the two adjacent lakes, L1 and L2, measured during the study period. Values are reported in g/L, with a measurement uncertainty of approximately ±3%.

The temperature, pH, and EC, all essential physicochemical parameters, were continuously monitored on-site. The monthly variations in the pH and EC throughout the study period are depicted in Figure 1, and salinity in Figure 2.

The water temperature in the studied period varied between 19 °C and 33 °C (measured at 1 m depth), and the values were strongly fluctuating, influenced either by the weather (rainy and cloudy days) or the tourist influx. The pH values of the lakes varied between 7.2 and 10.2 (±0.1) (Figure 1A,B), showing a larger interval compared to winter months (between October and April, the reported values were between 7.2 and 8.5 (±0.1) [7]. Specifically, the pH values in L1 were consistently higher than those in L2, both at the surface and at the depth layer. For L1 at 1 m depth, the pH showed little variation from May to August, from 8 to 8.4, with a significant peak of 9.8 in September, followed by a slight decrease to 8.9 in October. In L2 at 1 m depth, the pH varied from 7.2 in May, increased to 7.7 in June, and further to 8 in July and August. In September, there is a slight decrease to 7.9, further peaking to the maximum value of 8.1 in October. For the L1 surface, the pH was stable at around 8 from May to June, increased to 8.4 in August, significantly increased to 9 in September, and reached 10.2 in October. For the L2 surface, the pH was 7.5 in May, gradually increased to 7.8 in June, and then increased to 7.7 in July, followed by an increased value of 7.8 in August, 8 in September, and 9.5 in October. In summary, the pH levels of the two lakes were different, with L1 showing constantly higher values than L2 (with one exception, in October, for the depth of water), which was also observed in winter months [7], with one exception in April. Surface waters always showed more pronounced fluctuations and slightly higher values than those measured at 1 m depth.

The level of EC in water (Figure 1C,D) reflects its ability to conduct an electrical current, which is influenced by the concentration of dissolved salts and other inorganic substances such as chlorides, sulfates, sodium, calcium, and others. Higher concentrations of these substances lead to increased EC. Even small amounts of dissolved salts or chemicals can significantly raise the water’s conductivity levels. For L1, the EC levels showed different trends both in depth and at the surface. Measured values of EC at 1 m depth in L1 increased from 102 mS/cm in May to 114.6 mS/cm in June. Subsequently, it slightly decreased in July to 110.4 mS/cm before reaching 116.2 mS/cm in August. A slight decrease occurred in September (115.2 mS/cm), followed by a peak in October at 122.8 mS/cm. EC measured at the surface of L1 followed a different pattern, starting at 91.4 mS/cm in May. It increased to 110.5 mS/cm in June, peaked at 122.2 mS/cm in July, and then slightly decreased to 120 mS/cm in August. In September, it reached its highest value (123.4 mS/cm) and then dropped to 120 mS/cm in October. Depth and surface conductivity levels remained close and stayed elevated until October. The EC values measured in L2 depth and surface also fluctuated over the warm season. EC in L2 depth started at 118.8 mS/cm in May, decreased to 111.1 mS/cm in June, and peaked at 122.2 mS/cm in July. It showed a small drop to 116 mS/cm in August and then stabilized between 114.9 mS/cm in September and 119.7 mS/cm in October. The EC in L2 surface started at 105 mS/cm in May, decreased to 113.5 mS/cm in June, reached a peak at 120.8 mS/cm in July, slightly decreased to 116.1 mS/cm in August, and stayed around 114.3 mS/cm in September before reaching 115.5 mS/cm in October.

Overall, the EC values in both lakes increased from May to October, with some fluctuations. Depth levels were higher than surface levels from May to June, but this trend reversed from July to October, due to increased human activity combined with increased microbial activity specific to the warm season, suggesting elevated salt levels at the surface during summer months. Notably, L1 exhibited higher surface EC in July and August compared to L2, while L2 had higher depth EC values during the summer. Compared to EC values measured in winter months [7], when close values were measured in both lakes at 1 m depth, ranging between 110 and 120 mS/cm, and surface water values were substantially smaller, it appears that summer months are associated with higher EC values.

### 3.2. Raman Spectra of Raw Waters from the Two Lakes of Cojocna Balneary Resort During Summer Months from May 2023 to October 2023

**Figure 3:**
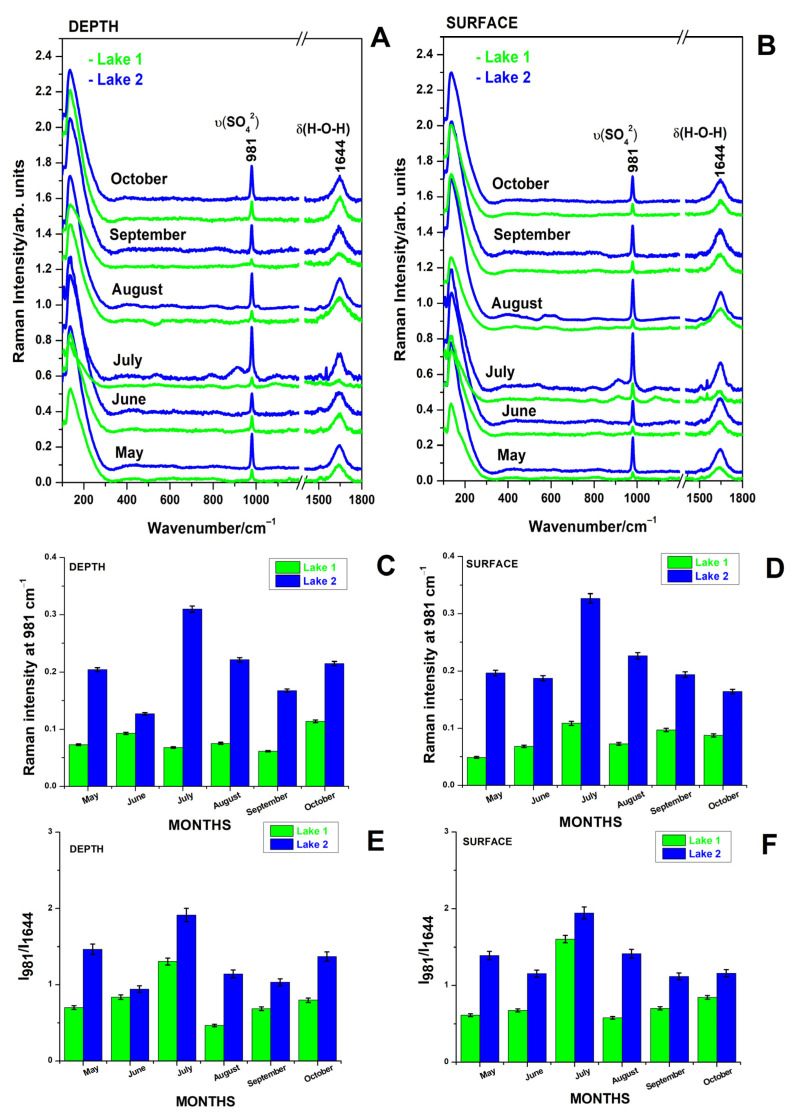
Normal Raman spectra collected from raw water samples of the lakes L1 and L2, at 1 m depth (**A**) and surface (**B**) during the summer monitoring campaign (May–October 2023). Panels (**C**,**D**) illustrate monthly variations in the Raman sulfate band intensity at 981 cm^−1^ for L1 (green) and L2 (blue), measured at 1 m (**C**) and surface level (**D**). The relative sulfate contribution was further evaluated using the Raman intensity ratio between the 981 cm^−1^ sulfate band and the water O–H bending mode at 1644 cm^−1^, highlighting monthly salinity dynamics at depth (**E**) and surface (**F**). Error bars are expressed as percentages of the measured values. Y axis offset was applied to spectra in panels (**A**,**B**) for improved visualization. Excitation: 532 nm.

Raman spectra of the raw water samples from lakes L1 and L2, collected from both depth and surface levels over the warm months between May and October 2023, are shown in Figure 3A,B. Prominent peaks were observed at 981 cm^−1^, assigned to the symmetric stretching mode of sulfate, ν (SO_4_^2−^) [9,13], which serves as a Raman spectral indicator of salinity and at 1644 cm^−1^, associated with the bending mode of water, δ(H−O−H)). Figure 3C,D illustrate the monthly variations in sulfate band intensity, while Figure 3E,F show the relative intensity of the sulfate to water band. For L2, this ratio remained consistently >1, whereas for L1, it was generally <1, except in July. These changes reflect the salinity dynamics in the hypersaline lakes, as sulfate is the only detectable contributor via normal Raman spectroscopy of raw waters. Depth measurements show higher sulfate concentrations in L2 throughout the observation period, with a notable peak in July and decreasing thereafter, while L1 remained relatively stable, with a slight increase in October. Surface measurements displayed a similar trend, with L2 exhibiting higher sulfate band intensity than L1, particularly in July, followed by declines in both lakes, though L1 showed a minor rebound in October. L2 consistently exhibited higher sulfate concentrations than L1, a pattern also observed during winter months [7]. Overall, sulfate levels in both lakes increased during the summer months due to evaporation, with a peak between May and July, and a partial recovery in October, reflecting seasonal variations.

### 3.3. SERS Spectra of Raw Waters from the Two Lakes at the Cojocna Balneary Resort During Summer Months from May 2023 to October 2023

The AgNPs used in the SERS experiments were routinely characterized by electron microscopy techniques, UV–VIS spectroscopy, and Raman analysis to ensure their consistency and performance as previously described [7]. The Raman spectrum of the blank colloid revealed only a weak water band, as expected.

**Figure 4:**
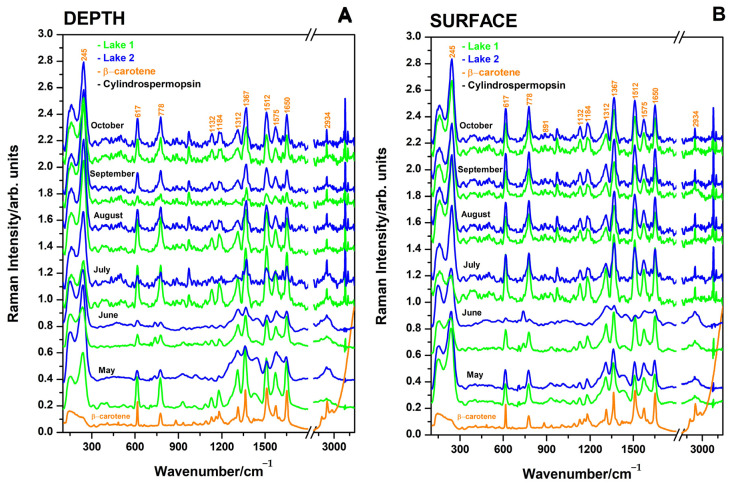
Typical SERS spectra collected from the depth (**A**) and surface waters (**B**) of L1 and L2 over the six-month summer period, as indicated on each spectrum. The bottom spectrum (orange line) is the SERS spectrum of pure β-carotene dissolved in ethanol at sub-micromole concentration (0.27 µM). Excitation: 532 nm^−1^.

SERS spectra were acquired from raw water samples collected at the surface and at 1 m depth in both hypersaline lakes (Figure 4). Each spectrum represents the average of three independent measurements performed on subsamples collected from fresh monthly samples. The SERS band positions and relative intensities showed reproducible main bands across both lakes, and this feature was similar to SERS spectra reported for winter months [7]; however, absolute SERS intensities varied month to month, even under identical experimental conditions. In addition, several spurious bands occurred in a few cases. The SERS analysis of the water samples revealed a consistent and characteristic signal attributed to β-carotene at sub-micromole concentrations [9,16], indicating the presence of cyanobacteria [17] near or attached to the AgNP aggregates. Peaks labeled orange correspond to SERS bands of chemisorbed β-carotene to the AgNP surface (1650, 1575, 1512, 1367, 1312, 1184, 1132, 1089, 778, and 617 cm^−1^) [15] and were visible in all SERS spectra from raw waters across months, though their intensities varied. The observed β-carotene SERS bands result from the aggregation of AgNP clusters instantly induced by salt ions from water. These AgNP clusters come into close contact with cyanobacteria that are rich in β-carotene. Given that a single cyanobacterium contains a small amount of β-carotene, the overall SERS signal reflects numerous interactions between AgNP aggregates and individual bacteria, thus indicating an indirect link between SERS intensity and cyanobacteria population. An increased cyanobacteria population leads to more intense β-carotene SERS bands, indicating that higher band intensities correlate with higher cyanobacteria abundance. For the reference SERS spectrum of pure β-carotene dissolved in ethanol, a fresh stock solution (purchased from Merck, Burlington, MA, USA) was used to prepare the SERS sample using the same AgNPs, by adding 10 µL of β-carotene-ethanol solution to 500 µL of AgNP colloidal solution. The β-carotene final SERS solution concentration was 0.27 µM. The changes in SERS band intensities could reflect factors determining cyanobacteria concentration, such as nutrient levels, sunlight exposure, and warm-season temperatures across seasons.

The SERS spectra measured from depth waters (Figure 4A) for both lakes exhibit noticeable variations in peak intensities across the months, reflecting seasonal changes in the lake chemistry. Like the spectra from depth waters, the SERS spectra collected from surface waters (Figure 4B) showed variations in peak intensities for both lakes, with some months showing increased intensities that correlate with increased β-carotene. Across both depth and surface waters, L2 (blue) often shows higher intensities at certain wavenumbers compared to L1 (green), indicating higher concentrations of specific compounds or differing lake environments. Notably, L1 consistently exhibits sharp peaks, whereas the spectra of L2 are broadened in May and June, likely due to the higher ionic strength and sulfate concentration in L2, which can induce spectral broadening through increased matrix interactions.

**Figure 5:**
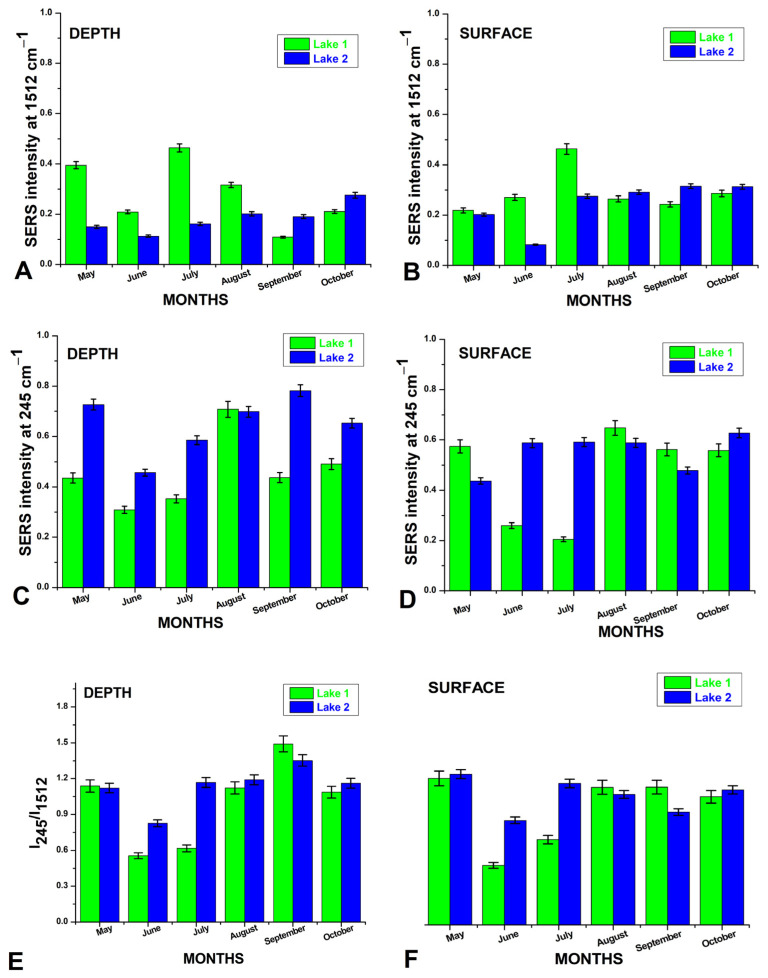
Comparative plot of the carotenoid SERS intensity band at 1512 cm^−1^ from waters of the two lakes, L1 (green) and L2 (blue), from 1 m depth (**A**) and surface (**B**); the Ag-Cl band intensity at 245 cm^−1^ for 1 m depth (**C**) and surface (**D**); and their ratio (**E**,**F**), respectively, during summer months from May 2023 to October 2023, as indicated. The calculated ratios of the SERS bands showed the variation in Cl-ions induced aggregation of AgNPs and concentration of carotenoids from cyanobacteria and reflects the monthly inorganic/organic balance of salt lakes in depth (**E**) and in surface waters (**F**). Error bars represent a percentage of data values for each lake’s depths and surface waters. Excitation: 532 nm.

Figure 5A–F show bar plots of SERS intensity of key bands and intensity ratios at specific wavenumbers for L1 and L2 over the six summer months. These figures illustrate how seasonal changes affect the Raman and SERS signals for different wavenumbers in each lake, with clear monthly patterns and differences between lakes. In the presence of salt ions in water, typical AgNP aggregation displays a strong SERS band at 245 cm^−1^, attributed to Ag-Cl adsorption [16], with signal intensity correlating with lake salt ion concentrations. The SERS technique was anticipated to reveal information about organic molecules in water—such as metabolites released by microalgae, cosmetic residues, pollutants, and other molecular species [18,19,20]. The intensity of the carotenoid SERS band at 1512 cm^−1^ is shown in Figure 5A,B for each investigation month, comparing water sampled from 1 m depth (Figure 5A) and the surface (Figure 5B). Additionally, the intensity of the Ag-Cl SERS band at 245 cm^−1^ is depicted in Figure 5C,D for both depths: 1 m (Figure 5C) and surface (Figure 5D). Variations in the 245 cm^−1^ Ag-Cl band reflect changes in chloride ion concentration, indicating higher or lower AgNP aggregation. Ratios of the SERS band intensities in the water spectra (Figure 5E,F) illustrate the monthly shifts in Cl^−^ concentration relative to organic carotenoids (from cyanobacteria), reflecting the inorganic-to-organic balance in salt lakes.

The SERS results suggested that Lake 1 often has higher intensities at 1512 cm^−1^, particularly in July for both depth and surface, than L2. Monthly variations in intensity may indicate changes in specific compounds or environmental conditions influencing the Raman-active species. Figure 5C,D show that Lake 2 sometimes has higher intensities, with noticeable peaks for both lakes in June and September. The variations between depth and surface measurements suggest that environmental factors affecting SERS intensity may differ by lake depth. The intensity ratio is an indicator of the relative concentrations of compounds responsible for these specific SERS peaks. The higher ratios observed in September, particularly for Lake 2 at depth, suggest seasonal changes in compound concentrations or matrix effects impacting SERS signals. The higher carotenoid-to-sulfate ratios observed in L1 during June and July are likely due to its lower sulfate and salinity levels, creating a more favorable environment for photosynthetic organisms, whereas the higher salinity and sulfate concentrations in L2 may limit carotenoid production.

Higher conductivity in both lakes (as seen in Figure 1C,D) during August also correlates with increased carotenoid activity. The elevated salinity could be due to increased evaporation, leading to a concentration of nutrients that support the blooming of photosynthetic organisms like cyanobacteria. Larger microorganisms such as Dunaliella species, although present in waters, seemed to be free of AgNPs on their surface, thus not contributing to the overall SERS signal as observed under point analyses under light microscopy and Raman microspectroscopy.

The elevated carotenoid bands in L1, especially at the surface, are also linked to the lower sulfate and salinity levels observed in the Raman spectra, suggesting that L1 is a more favorable environment for these organisms during the summer months. The presence of β-carotene and other carotenoids indicates healthy populations of photosynthetic organisms in the lakes. These organisms are a vital part of the aquatic ecosystem, contributing to the food web and helping maintain water quality through oxygen production and nutrient cycling. However, excessive biological activity, especially in combination with high salinity, can also lead to algal blooms, which may degrade water quality over time. This could be a point to monitor if there is a concern about eutrophication or the excessive growth of algae in these lakes, particularly in L1. The higher carotenoid-to-sulfate ratios observed in L1 during June and July can be attributed to its lower sulfate and salinity levels, which create a more favorable environment for photosynthetic organisms. This contrasts with L2, where higher salinity and sulfate concentrations may limit carotenoid production. These differences align with the elevated carotenoid bands in L1, indicating healthy populations of photosynthetic organisms and active ecosystem dynamics.

**Figure 6:**
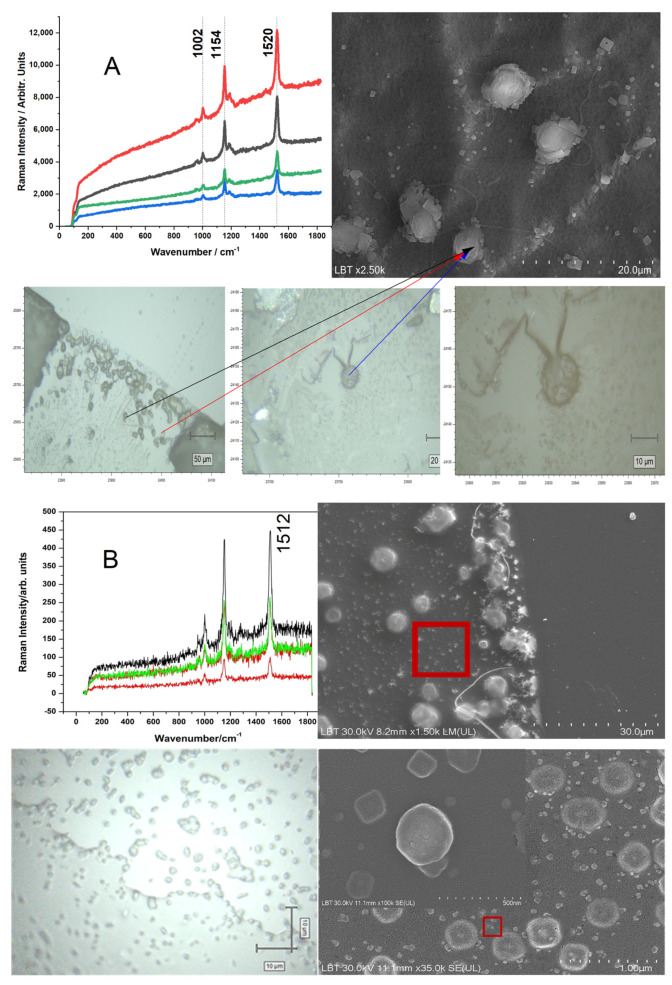
Resonance Raman spectra recorded from drop-coated raw water droplets, highlighting the strong spectral signature of the microalga *Dunaliella salina* (**A**) and cyanobacteria (**B**). The spectra are shown with different colored lines to distinguish between replicate measurements from individual droplets and to highlight spectral reproducibility. Red boxes indicate the characteristic Raman bands that are diagnostic for *Dunaliella salina* or cyanobacteria. The presence of *Dunaliella salina* and cyanobacteria is confirmed by corresponding optical micrographs captured with the video camera of the Raman microscope, alongside a representative SEM image highlighting the characteristic morphology of these algae. Scale bar: (**A**): 20 µm in SEM image and 50, 20, and 10 µm in optical micrographs, respectively, with the arrows indicating the optically spotted *Dunaliella salina* in the sample and their SEM; (**B**): 30 µm (upper SEM), 10 µm (optical micrograph), and 1 µm (lower SEM, with inset detail at 500 nm).

The SERS signal of β-carotene comes from microorganisms present in the lakes (Figure 6). Both light microscopy and SEM revealed the presence of *Dunaliella salina* (Figure 6A) and cyanobacteria (Figure 6B). Larger *Dunaliella salina* (~10 μm) and cyanobacteria (~1 μm) give different Raman spectra associated with carotenoids. While *Dunaliella salina* shows a C=C bond vibration at 1520 cm^−1^ (Figure 6A), cyanobacteria exhibit the same bond vibrating at 1509 cm^−1^ (Figure 6B), suggesting the presence of different carotenoid compositions from these waters. A peak occurs at 1511 cm^−1^, which results from cyanobacteria, reflecting a shift in the peak position as a result of silver nanoparticles attaching to cyanobacteria (Figure 6B).

Both light microscopy and SEM revealed the presence of *Dunaliella salina* (Figure 6A) and cyanobacteria (Figure 6B). Larger *Dunaliella salina* (~10 µm) and cyanobacteria (~1 µm) give different Raman spectra associated with carotenoids. While *Dunaliella salina* shows a C=C bond vibration at 1520 cm^−1^ (Figure 6A), cyanobacteria exhibit the same bond vibrating at 1509 cm^−1^ (Figure 6B), suggesting the presence of different carotenoid compositions.

In summary, the results provide meaningful insights into the seasonal dynamics of salt lakes through Raman and SERS analyses. The Raman spectra highlight sulfate concentration changes reflecting salinity fluctuations, while SERS spectra reveal the presence and seasonal variation in β-carotene, attributed to cyanobacterial activity. The monthly intensity changes in β-carotene SERS bands across depth and surface levels, especially in Lake 2, suggested a direct link between lake chemistry, organic content, and environmental conditions, underscoring the value of these techniques for monitoring seasonal lake ecosystem changes.

### 3.4. X-Ray Diffraction (XRD) Analysis of Salt Baches

**Figure 7:**
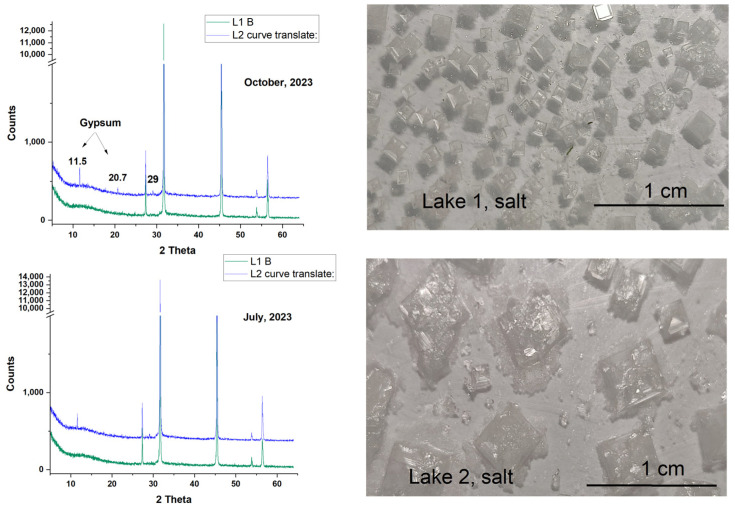
XRD pattern of the total salt resulted from room-temperature (20 °C) evaporation of 20 mL of surface water from lakes L1 and L2, respectively, in July and October 2023, as indicated. Micrographs of salt crystals over 2.5 × 1.6 cm surface shown on the right side are specific to each lake, as indicated.

Figure 7 presents the XRD patterns of total salts obtained through room-temperature (20 °C) evaporation of 20 mL surface water samples collected from lakes L1 and L2 in July and October 2023. The results revealed that small diffraction peaks corresponding to gypsum are particularly characteristic of the L2 samples. In contrast, the salts obtained from L1 water show the presence of halite only, as identified by XRD analysis. Regardless of these results, consistently higher sulfate concentrations for L2 samples cannot be explained by the presence of gypsum, given its poor water solubility. Moreover, the main salinity contributor in the lakes is halite.

## 4. Discussion

### 4.1. Statistical Correlation Between Physicochemical Properties and Raman and SERS Signals of Lakes

The Pearson correlation coefficient was employed to facilitate a comprehensive investigation of the relationship between the Raman and SERS spectral data of two lake water samples and their respective physicochemical parameters (EC, pH). This analysis aimed to elucidate how changes in relevant peak intensity within the Raman and SERS spectra correlate with fluctuations in pH and EC over a six-month timeline.

The Raman sulfate band at 981 cm^−1^ in spectral data shows variable trends and correlations with conductivity and pH values. As conductivity increases, reflecting higher salt and ion content, the intensity of the 981 cm^−1^ sulfate band also increases, confirming that sulfate concentration is a major contributor to the overall salinity of both lakes. Lake L2 shows higher sulfate levels and conductivity throughout the summer period, particularly in August and September, which aligns with its higher pH values. The increase in pH, together with high conductivity, suggests that L2 experiences more intense bedrock dissolution and salt accumulation, resulting in higher sulfate levels detected in the Raman spectra. In contrast, L1 shows lower conductivity and sulfate levels, along with relatively stable pH values, indicating less pronounced bedrock dissolution and salt accumulation, leading to lower salinity and weaker Raman sulfate signals.

**Figure 8:**
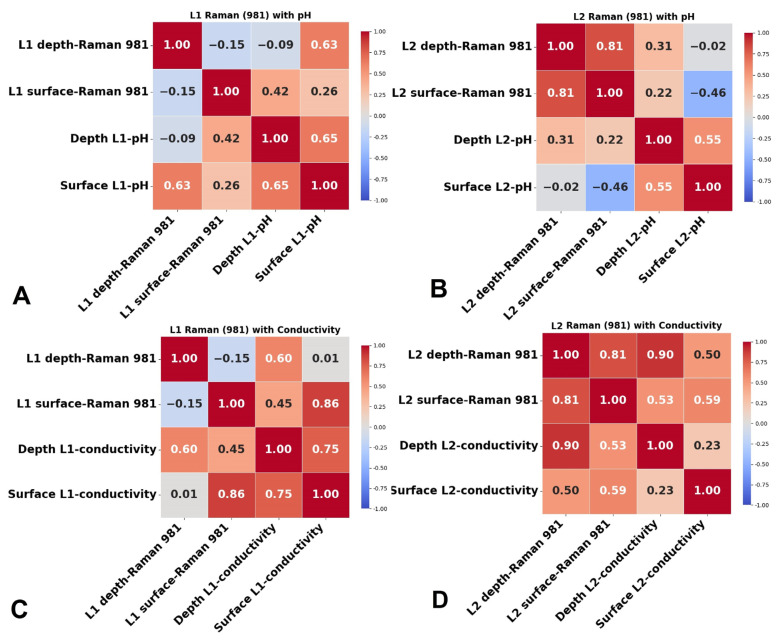
Representative correlation plots between datasets: (**A**) Correlation between Raman data and pH for L1 and (**B**) L2. (**C**) Correlation between Raman data and EC for L1 and (**D**) L2.

Figure 8 shows the correlation coefficients between the Raman sulfate signal at 981 cm^−1^ and the pH and EC measurements for both lakes. In L1, a moderate positive correlation is observed between the Raman sulfate signal and physicochemical parameters across surface and deep-water samples (Figure 8A,C). In L2, surface pH values show a slight negative correlation with the surface Raman signal, whereas deep-water Raman data display moderate positive correlation with both depth pH and conductivity, as well as with surface and depth conductivity (Figure 8B,D). Overall, each lake exhibits distinct correlation patterns: L2 shows a generally positive correlation between Raman signals and physicochemical parameters, while L1 presents a more complex relationship.

In L1, the surface pH and depth Raman sulfate band have a strong positive correlation (0.63), and depth pH and surface Raman data show a moderate correlation (0.42). Correlations between Raman data and conductivity show a moderate positive correlation between depth conductivity and both depth (0.6) and surface (0.45) Raman data, as well as a strong correlation (0.86) between surface conductivity and surface Raman data. Additionally, the correlation coefficient (0.65) between surface and depth pH values indicates synchronized pH changes, while a strong correlation (0.75) between the surface and depth of conductivity suggests consistent conductivity levels across depths. The varied correlation patterns in L1, especially the moderate-to-strong correlations between conductivity and Raman data at different depths, imply complex interactions between water chemistry and the sulfate-related Raman signal. This could indicate stratification within the lake or distinct chemical profiles at each layer, influenced by varying environmental or biological factors.

In L2, a moderate negative correlation (−0.46) exists between surface pH and surface Raman data at 981 cm^−1^, while depth pH and depth Raman data show a moderate positive correlation (0.31). The negative correlation between surface pH and Raman intensity at the surface might indicate a unique chemical interaction or condition at the lake’s surface, suggesting that increased acidity (lower pH) is associated with higher Raman signals, due to changes in sulfate ionization or interactions with surface organic matter. Positive correlations are seen between Raman data and conductivity, including a moderate correlation between surface Raman data and both depth (0.53) and surface (0.59) conductivity, a moderate correlation between surface conductivity and depth Raman (0.50), and a strong correlation (0.9) between depth Raman data and depth conductivity. Surface and depth pH levels also show a moderate positive correlation (0.55), indicating coordinated changes, and the Raman sulfate signal at 981 cm^−1^ demonstrates a high correlation (0.81) between depths, suggesting uniform environmental conditions across depth layers. In addition to the Pearson correlation coefficients, the corresponding *p*-values were evaluated, which are provided in the Supplementary Information (Figure S2).

In conclusion, both lakes show a positive correlation between conductivity and the Raman signal, especially in Lake L2, where depth conductivity and Raman data correlate strongly (0.9), suggesting that ion concentration or mineral content may significantly influence Raman intensity. In contrast, variable correlations in L1 may reflect dynamic ionic concentrations or conditions between surface and deep waters. These differences suggest that each lake may benefit from tailored monitoring approaches. For L2, consistent profiles across depths indicate that surface measurements could serve as proxies for deeper layers, whereas L1’s varied relationships imply that separate assessments at each depth may be necessary to fully capture its chemical and physical dynamics.

**Figure 9:**
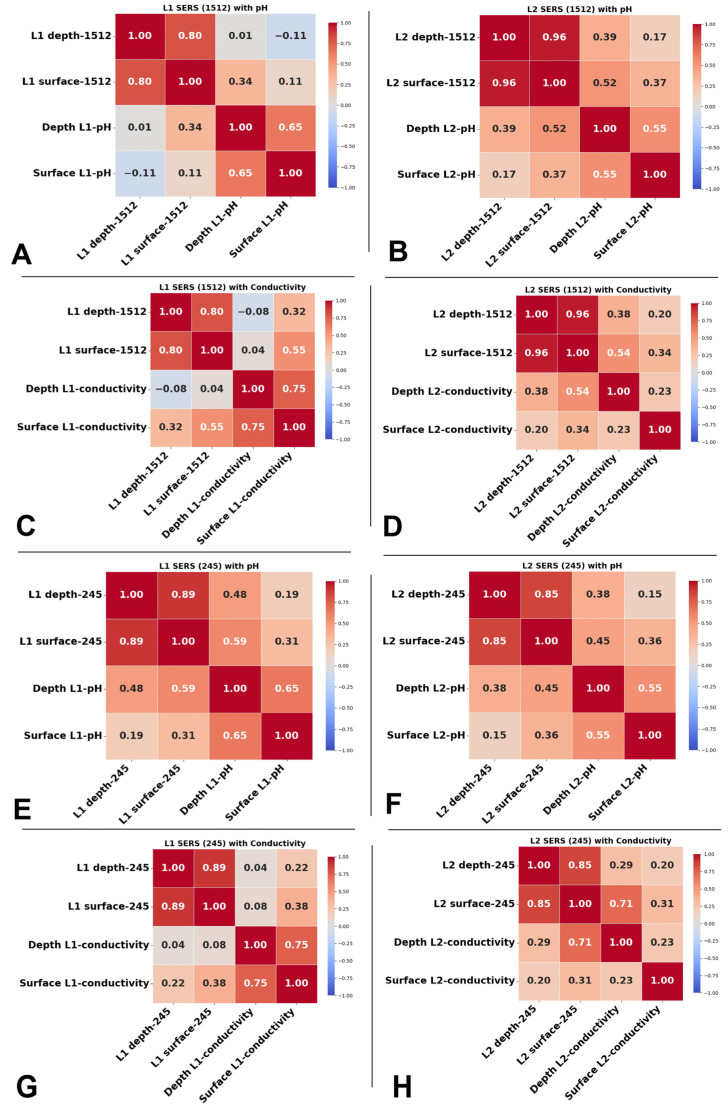
Representative correlation plots between datasets. Correlation between SERS spectral data at 1512 cm^−1^ and pH for (**A**) L1 and (**B**) L2; correlation between SERS spectral data at 1512 cm^−1^ and EC for (**C**) L1 and (**D**) L2; correlation between SERS spectral data at 245 cm^−1^ and pH (**E**) L1 and (**F**) L2. Correlation between SERS spectral data at 245 cm^−1^ and EC for (**G**) L1 and (**H**) L2.

Figure 9 displays the correlation coefficients between the SERS signals at 1512 cm^−1^ of the Cl^−^ band and at 1512 cm^−1^ of the β-carotene band, along with the measured pH and EC. The SERS band intensities from the spectral data show general positive moderate correlations with the physicochemical parameters in the summer period. In L1, a moderate positive correlation (0.34) is observed between the SERS signal at 1512 cm^−1^ from depth pH (Figure 9A), and a moderate positive correlation is observed between depth SERS data and surface EC (0.32) and depth EC (0.55) (Figure 9C). The SERS signal at 1512 cm^−1^ shows a high positive correlation (0.80) between depth and surface SERS, suggesting that SERS signals at different depths for this wavenumber are closely related. Additionally, there is a strong correlation (0.96) between surface and depth 1512 cm^−1^ spectral data, suggesting that SERS signals at different depths for this wavenumber are closely related.

In L2, a more positive correlation can be observed between the SERS band at 1512 cm^−1^ and pH and EC (Figure 9B,D). The correlation coefficient between the depth pH and the depth SERS band is 0.39, while that between the surface SERS band and the surface pH is 0.37, and that between the surface SERS band and the depth pH is 0.52. In L2, a strong positive correlation can be observed between SERS spectral data and EC, namely 0.38 for depth conductivity and depth SERS data, 0.54 for surface SERS data, and 0.34 between surface conductivity and surface SERS data. There is a strong correlation (0.96) between surface and depth 1512 cm^−1^ spectral data, suggesting that SERS signals at different depths for this wavenumber are closely related.

The correlation coefficients between physicochemical parameters and SERS band at 245 cm^−1^ were generally positive but weak to moderate. In L1, moderate correlations were observed between pH and the 245 cm^−1^ band at depth (0.48) and at the surface (0.59), and a weaker correlation was found between the surface pH and the surface 245 cm^−1^ band (0.31). Surface conductivity also showed a weak positive correlation with the 245 cm^−1^ band (0.38), while no correlation was observed between depth conductivity and the 245 cm^−1^ band (Figure 9G). In L2, both surface and depth conductivity showed only weak correlations with the 245 cm^−1^ band (0.20 and 0.29, Figure 9H). Importantly, the SERS signal at 245 cm^−1^ showed strong correlations between surface and depth samples in both lakes (0.89 in L1 0.89; 0.73 in L2), suggesting that this spectral feature is consistent across depths.

In L2, a moderate positive correlation can be observed between depth pH and SERS intensity at 245 cm^−1^, denoted “depth 245” (0.38) and “surface 245” (0.45), as well as between surface pH and surface 245 (0.36). The correlation coefficient between conductivity and 245 SERS spectral data indicates a moderate positive correlation between surface conductivity and surface 245, and a strong correlation between depth conductivity and surface 245.

The correlation coefficient between surface and depth pH values was 0.55 in L1 and 0.65 in L2, suggesting that pH levels at different depths tend to change in the same direction, indicating a consistent pH profile in L1 and L2. Additionally, there is a strong correlation (0.75) in L1 between surface and depth conductivity, indicating that conductivity levels across depths tend to vary together, potentially suggesting a consistent conductivity profile within L1. In addition to the Pearson correlation coefficients, the corresponding *p*-values were calculated and are presented in the Supplementary Information (Figure S3). The correlation analysis reveals that SERS signals at 1512 cm^−1^ and 245 cm^−1^ show moderate-to-strong positive correlations with pH and EC in both lakes during the summer period. Notably, strong correlations were observed between surface and depth SERS signals (up to 0.96), indicating consistent spectral responses across depths. In Lake L1, SERS at 1512 cm^−1^ showed moderate correlations with pH and EC, while in Lake L2, stronger correlations were noted, particularly between SERS data and depth EC. Similarly, for the 245 cm^−1^ band, moderate correlations with pH and EC were found in both lakes, with a strong depth EC correlation in L2. Additionally, surface and depth pH and EC values exhibited positive correlations, suggesting uniform physicochemical profiles across depths in both lakes.

### 4.2. Correlation Between Salinity and Conductivity of Lakes

**Figure 10:**
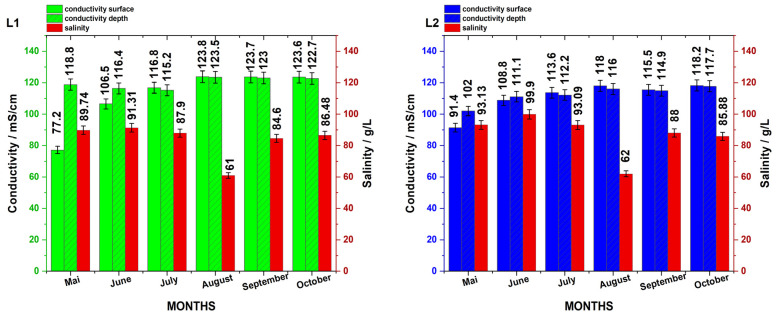
Comparative double plot of conductivity and salinity values during the investigated period in the hypersaline lakes L1 and L2. Conductivity is expressed in mS/cm, while salinity is expressed in g/L with error bars of 3%.

Measured values of salinity and EC in the Cojocna lakes showed no statistically significant correlation. Consequently, salinity measurements were excluded from further statistical analyses. Figure 10 illustrates the fluctuations in salinity in relation to both surface and depth conductivity over the sampling period.

This lack of correlation is not unexpected, as the previous literature has reported that at high salinity levels, the relationship between salinity and conductivity becomes non-linear and complex due to ion interactions and changes in water composition.

For comparison, earlier measurements of these lakes’ water properties were reported by Czellecz et al. (2016) [21], who measured salinity and EC using a Thermo Orion Star, Thermo Fisher Scientific, Waltham, MA, USA, portable multimeter in March–April 2015. Their data indicated total dissolved solids (TDSs) of approximately 25 g/L and EC values around 49–50 mS/cm, with salinity values of 31.4 and 31.2 ppt, respectively. These historical values contrast markedly with the present in situ measurements from 2023, suggesting significant changes in the lakes’ physicochemical parameters over the past eight years.

The lack of correlation between salinity and EC in hypersaline lakes has been previously discussed [15,16,17,18] and can have many causes, such as changes in ion composition, ion interactions, precipitation of certain salts at high concentrations, and reduced ion mobility due to increased viscosity, further due to microbial abundance and their metabolites. Although EC is a useful proxy for salinity in normal waters, it becomes unreliable in extreme hypersaline conditions unless calibrated specifically for the lake’s chemistry. Alcorlo et al. (1996) [22] showed that there are limitations in predicting salinity from conductivity. They analyzed 128 samples from 69 Iberian salt lakes and found that although a log–log regression model yielded an R^2^ ≈ 0.88, the predictive power was poor, primarily due to the heterogeneous ionic composition across lakes, undermining a universal EC-to-salinity conversion. Another study [23] reported no correlation between specific conductivity and TDS in the case of saline lakes in Alberta and Saskatchewan, which exhibit a wide range of chemical and physical properties, including variations in salinity, ionic composition, and stratification. These lakes are typically athalassohaline, meaning their ionic composition differs from seawater, with sulfates being the dominant anion in most cases. In such athalassohaline lakes, the dominant anion was sulfate (SO_4_^2−^) rather than chloride, resulting in a non-1:1 ion charge ratio and breaking the usual relationship [23]. Furthermore, the standard salinity/conductivity units become invalid at extreme salinity; high precision demands alternative measures, as highlighted by Anati (1999) [24]. Finally, a review [18] on the geochemical uniqueness of hypersaline lakes, including how distinct ionic profiles and thermodynamics (precipitation, ion pairing, etc.) alter salinity–conductivity behavior, highlighted how cutting-edge technologies can provide new insights into the study of hypersaline ecology and shed light on these understudied ecosystems [25].

## 5. Conclusions

This work highlights how Raman and SERS techniques can help us better understand the seasonal and depth-related changes in hypersaline lakes. Our results showed that sulfate bands detected by Raman, especially around 981 cm^−1^, follow seasonal patterns, caused by evaporation and increased salinity during summer, especially in Lake L1. In contrast, Lake L2 had more stable conditions over time. The SERS signals, particularly those at 1512 cm^−1^ (linked to β-carotene from cyanobacteria) and 245 cm^−1^ (Ag–Cl), gave us important clues about both the inorganic content and biological activity in the lakes. These signals showed moderate-to-strong positive correlations with pH and conductivity, especially during the summer months. The high correlation between surface and depth SERS signals in Lake L2 suggests a more uniform chemical profile, while Lake L1 showed more differences between layers, meaning depth-specific measurements are needed there. Although salinity and conductivity did not correlate statistically, as expected, their seasonal trends supported our spectral observations. Overall, combining Raman and SERS with basic physicochemical measurements offers a useful way to track changes in saline lakes, and it could be helpful for monitoring environmental health or managing the therapeutic use of such lakes in the future.

The findings of this research could have significant implications for both the conservation of these natural resources and the optimization of their use in therapeutic applications. Through this pilot SERS monitoring study, we provided meaningful insights into the organic–inorganic balance and dynamics of hypersaline waters, expanding our knowledge of salt lake environments during the warm season and their potential use in balneotherapy, wellbeing, and touristic activities for health benefits. The outcomes will not only underscore the importance of using advanced analytical Raman and SERS techniques in environmental science but also pave the way for future comprehensive studies or continuous monitoring efforts in this field.

## Data Availability

Data is contained within the article.

## Supplementary Materials

The following supporting information can be downloaded at: https://www.mdpi.com/article/10.3390/bios15100655/s1, Figure S1: Transmission electron microscopy (TEM) image of silver nanoparticles (AgNPs) showing their spherical shape and uniform distribution; Figure S2: *p*-value heatmaps corresponding to the pairwise correlations shown in Figure 8. A–D represent the same variable groups as in the correlation heatmaps. The color scale ranges from 0 (statistically significant, darker shades) to 1 (non-significant, lighter shades), indicating the reliability of the observed correlations; Figure S3: *p*-value heatmaps corresponding to the pairwise Pearson correlations between datasets. *p*-values for the correlation be-tween SERS spectral data at 1512 cm^−1^ and pH for (A) L1 and (B) L2; *p*-values for the correlation between SERS spectral data at 1512 cm^−1^ and EC for (C) L1 and (D) L2; *p*-values for the correlation between SERS spectral data at 245 cm^−1^ and pH for (E) L1 and (F) L2; *p*-values for the correlation between SERS spectral data at 245 cm^−1^ and EC for (G) L1 and (H) L2.

## References

[1] Tran N.H., Li Y., Reinhard M., Goh K.C., Sukarji N.H.B., You L., He Y., Gin K.Y.H. (2020). Quantification of Cylindrospermopsin, Anatoxin-a and Homoanatoxin-a in Cyanobacterial Bloom Freshwater Using Direct Injection/SPE Coupled with UPLC-MS/MS. Sci. Total Environ..

[2] Baricz A., Levei E.A., Șenilă M., Pînzaru S.C., Aluaş M., Vulpoi A., Filip C., Tripon C., Dădârlat D., Buda D.M. (2021). Comprehensive Mineralogical and Physicochemical Characterization of Recent Sapropels from Romanian Saline Lakes for Potential Use in Pelotherapy. Sci. Rep..

[3] Ionescu E.V., Suta M., Surdu O., Oprea C., Stoicescu R.M., Taralunga G.L.G. (2014). Clinical and Biological Effects Induced by Sapropelic Mud from the Lake Techirghiol in Patients with Osteoarthritis. J. Environ. Prot. Ecol..

[4] Katz U., Shoenfeld Y., Zakin V., Sherer Y., Sukenik S. (2012). Scientific Evidence of the Therapeutic Effects of Dead Sea Treatments: A Systematic Review. Semin. Arthritis Rheum..

[5] Şerban G., Alexe M., Touchart L. (2005). Morphological Evolution and Salinity of Cojocna Lakes (Transylvanian Plain, Romania). Bull. d’Assoc. Geogr. Fr..

[6] Krézsek C., Filipescu S. (2005). Middle to Late Miocene Sequence Stratigraphy of the Transylvanian Basin (Romania). Tectonophysics.

[7] Molnár C., Drigla T.D., Barbu-Tudoran L., Bajama I., Curean V., Cîntă Pînzaru S. (2023). Pilot SERS Monitoring Study of Two Natural Hypersaline Lake Waters from a Balneary Resort during Winter-Months Period. Biosensors.

[8] Li Z., Wang J., Li D. (2016). Applications of Raman Spectroscopy in Detection of Water Quality. Appl. Spectrosc. Rev..

[9] Cinta Pinzaru S., Ardeleanu M., Brezestean I., Nekvapil F., Venter M.M. (2019). Biogeochemical Specificity of Adjacent Natural Carbonated Spring Waters from Swiss Alps Promptly Revealed by SERS and Raman Technology. Anal. Methods.

[10] Maškarić K., Cîntă Pînzaru S., Dumitru D.-A., Molnar C. Raman Spectroscopy Techniques and Technology as a Tool in Environmental Water Analysis. Proceedings of the 2024 “Air and Water—Components of the Environment” Conference Proceedings.

[11] Lee P.C., Meisel D. (1982). Adsorption and Surface-Enhanced Raman of Dyes on Silver and Gold Sols. J. Phys. Chem..

[12] Wei T., Simko V. (2017). R Package “Corrplot”: Visualization of a Correlation Matrix (Version 0.84). https://github.com/taiyun/corrplot.

[13] Brezestean I., Nekvapil F., Cinta Pinzaru S. Analysis of Hypersaline Waters from Cojocna Balneary Resorts (Romania) Using Raman Spectroscoy Techniques. Proceedings of the Conference “Air and Water Components of the Environment”.

[14] Müller Molnár C., Cintă Pînzaru S., Chis V., Feher I., Glamuzina B. (2023). SERS of Cylindrospermopsin Cyanotoxin: Prospects for Quantitative Analysis in Solution and in Fish Tissue. Spectrochim. Acta Part A Mol. Biomol. Spectrosc..

[15] Cintə Pinzaru S., Müller C., Tomšic S., Venter M.M., Cozar B.I., Glamuzina B. (2015). New SERS Feature of β-Carotene: Consequences for Quantitative SERS Analysis. J. Raman Spectrosc..

[16] Bell S.E.J., Sirimuthu N.M.S. (2005). Surface-Enhanced Raman Spectroscopy as a Probe of Competitive Binding by Anions to Citrate-Reduced Silver Colloids. J. Phys. Chem. A.

[17] Pinzaru S.C., Csilla M.M., Ioana B., Glamuzina B. (2016). Cyanobacteria Detection and Raman Spectroscopy Characterization with a Highly Sensitive, High Resolution Fiber Optic Portable Raman System. Stud. Univ. Babes Bolyai Phys..

[18] Pinzaru S.C., Müller C., Ujević I., Venter M.M., Chis V., Glamuzina B. (2018). Lipophilic Marine Biotoxins SERS Sensing in Solutions and in Mussel Tissue. Talanta.

[19] Müller C., Glamuzina B., Pozniak I., Weber K., Cialla D., Popp J., Cîntǎ Pînzaru S. (2014). Amnesic Shellfish Poisoning Biotoxin Detection in Seawater Using Pure or Amino-Functionalized Ag Nanoparticles and SERS. Talanta.

[20] Josefson M., Walsh A., Abrahamsson K. (2015). Imaging and Identification of Marine Algal Bioactive Compounds by Surface Enhanced Raman Spectroscopy (SERS). Natural Products From Marine Algae, Methods in Molecular Biology.

[21] Czellecz B., Gabor I., Ravasz L., Schiopu G., Szopos N. (2016). Saline Water Resurces in Cluj-Napoca Surroundings. Air Water Compon. Environ..

[22] Alcorlo P., Baltanás A., Montes C. (1996). Is It Possible to Predict the Salinity of Iberian Salt Lakes from Their Conductivity?. Hydrobiologia.

[23] Bowman J.S., Sachs J.P. (2008). Chemical and Physical Properties of Some Saline Lakes in Alberta and Saskatchewan. Saline Syst..

[24] Anati D.A. (1999). The Salinity of Hypersaline Brines: Concepts and Misconceptions. Int. J. Salt Lake Res..

[25] Saccò M., White N.E., Harrod C., Salazar G., Aguilar P., Cubillos C.F., Meredith K., Baxter B.K., Oren A., Anufriieva E. (2021). Salt to Conserve: A Review on the Ecology and Preservation of Hypersaline Ecosystems. Biol. Rev..

