# Supplementary material for: SERS Detection of Environmental Variability in Balneary Salt Lakes During Tourist Season: A Pilot Study
Source: Biosensors (Basel). 2025 Oct 1;15(10):655. doi: 10.3390/bios15100655 (PMC12564343; doi:10.3390/bios15100655)
Supplement: Supplementary file 1 [file biosensors-15-00655-s001.zip › biosensors-3807342-supplementary.pdf]

biosensors

MDPI

Article

SERS Detection of Environmental Variability in Balneary Salt Lakes During Tourist Season: A Pilot Study

Csilla Molnár [1,2,3*], Karlo Maškarić[2,3], Lucian Barbu-Tudoran [4], Tudor Tamas[5], Ilirjana Bajama [2,3] and Simona Cîntă Pînzaru [2,3] *

[1] National Institute for Research and Development of Isotopic and Molecular Technologies, 67-103 Donath, 400293 Cluj-Napoca, Romania
[2] Biomolecular Physics Department, Babeş-Bolyai University, Kogălniceanu 1, 400084 Cluj Napoca, Romania
[3] Institute for Research, Development and Innovation in Applied Natural Sciences, Babes-Bolyai University, Fantanele 30, 400327 Cluj-Napoca, Romania
[4] Electron Microscopy Centre, Babeș-Bolyai University, Clinicilor 5-7, 400006 Cluj-Napoca, Romania; lucian.barbu@itim-cj.ro
[5] Geology Department, Babeş-Bolyai University, Kogălniceanu 1, 400084 Cluj Napoca, Romania
* Correspondence: csilla.molnar@itim-cj.ro (C.M.); simona.pinzaru@ubbcluj.ro (S.C.P.)

Transmission electron microscopy (TEM) coupled with energy dispersive X-ray spectroscopy (EDX) was used to characterize the size and morphology of the silver nanoparticles (AgNPs) (Fig. S1). Measurements were performed using a Hitachi SU8230 cold field emission scanning transmission electron microscope (Hitachi, Japan).

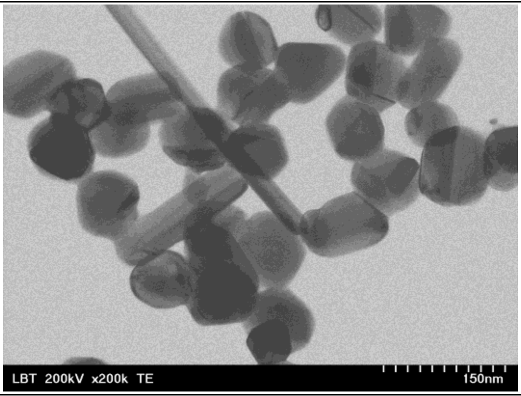

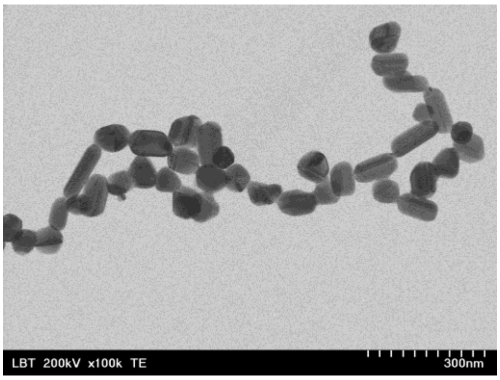

Figure S1. Transmission electron microscopy (TEM) image of silver nanoparticles (AgNPs) showing their spherical shape and uniform distribution.

The TEM images (Figure S1) revealed that the AgNPs were spherical and uniformly distributed. This stock of AgNPs was used for all the SERS measurements, providing a consistent substrate for signal enhancement. The uniform size and shape of the nanoparticles are expected to contribute to reproducible SERS enhancement across different measurements.

P-value correlation analysis.

Pairwise statistical tests were conducted to evaluate the significance of correlations among the selected variables. The resulting p-value heatmaps (Fig. S2) provide an overview of the reliability of the observed associations.

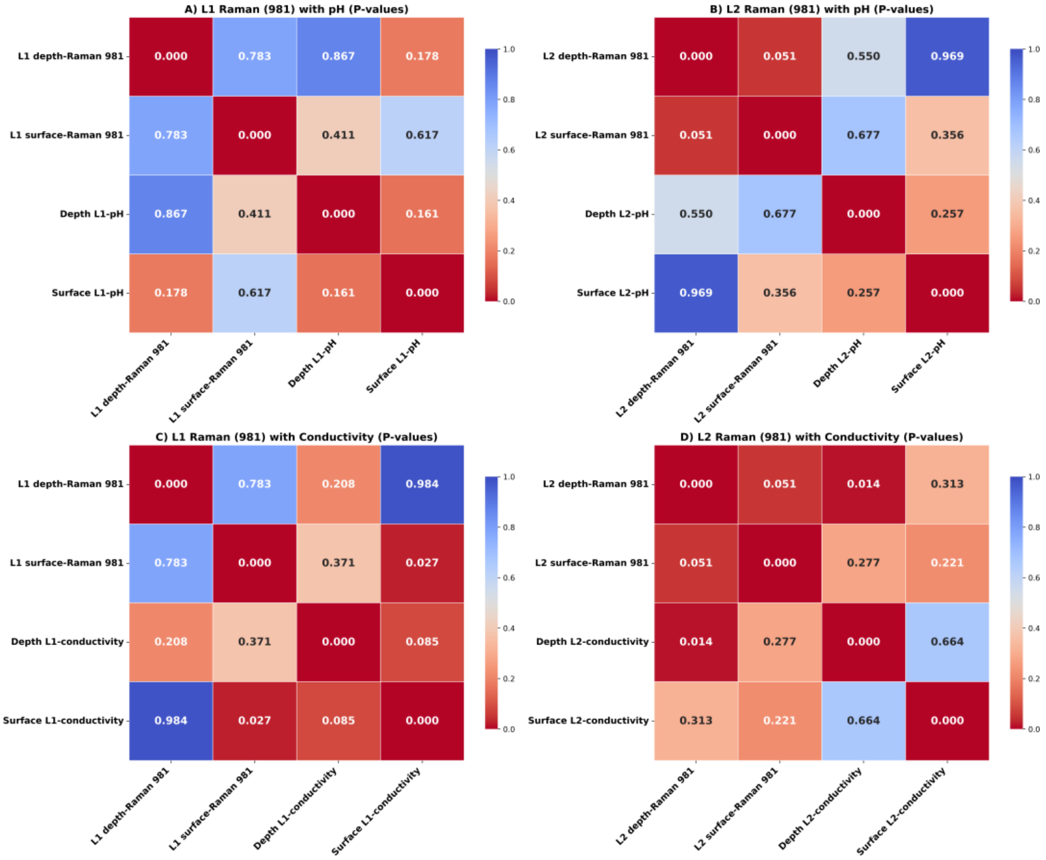

Figure S2. P-value heatmaps corresponding to the pairwise correlations shown in Figure 8. A–D represent the same variable groups as in the correlation heatmaps. The color scale ranges from 0 (statistically significant, darker shades) to 1 (non-significant, lighter shades), indicating the reliability of the observed correlations.

To evaluate the statistical significance of the observed correlations between SERS spectral data and environmental parameters (Fig. S3), we calculated the pairwise Pearson correlation coefficients along with their corresponding p-values. Figure X presents the p-value heatmaps for all measured variables, highlighting the significance of correlations across different depths (L1 and L2) and spectral bands (1512 cm^{-1} and 245 cm^{-1}) for both pH and electrical conductivity. Lower p-values indicate stronger evidence that the observed correlations are statistically significant.

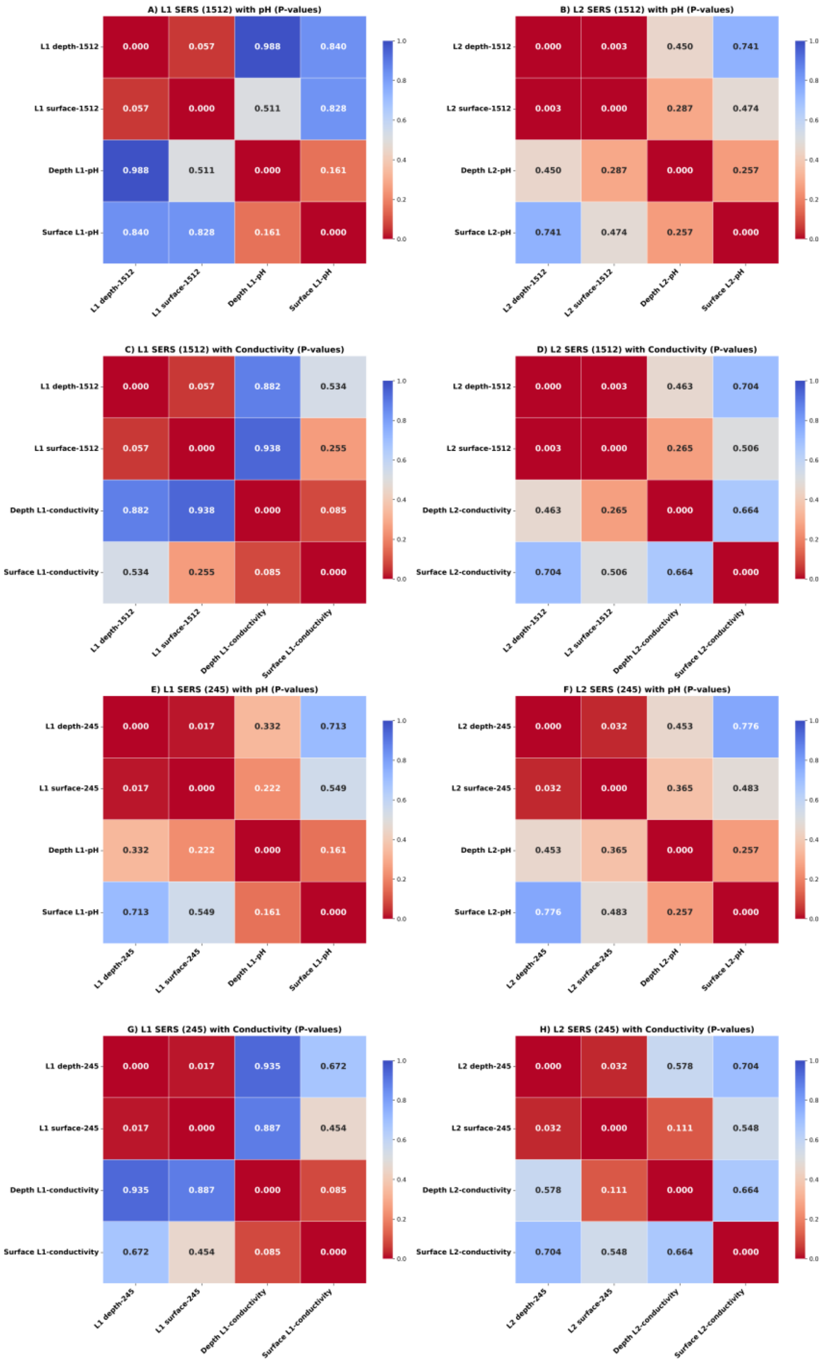

Figure S3. P-value heatmaps corresponding to the pairwise Pearson correlations between datasets. P-values for the correlation between SERS spectral data at 1512 cm^{-1} and pH for (A) L1 and (B) L2; p-values for the correlation between SERS spectral data at 1512 cm^{-1} and EC for (C) L1 and (D) L2; p-values for the correlation between SERS spectral data at 245 cm^{-1} and pH for (E) L1 and (F) L2; p-values for the correlation between SERS spectral data at 245 cm^{-1} and EC for (G) L1 and (H) L2.
